# Effect of Traditional Chinese Medicine External Treatment in Older Patients with Nocturia: A Meta-analysis: Short title: Traditional Chinese Medicine External Therapy for Elderly Nocturia

**DOI:** 10.31661/gmj.v15i.3986

**Published:** 2026-01-30

**Authors:** Huiying Zhang, Jinhua Geng, Chunyan Ruan, Qiaoli Lin, Liqin Chen, Yanxiang Luo, Shaoqing Liu, Shaoxia Wu

**Affiliations:** ^1^ Guangdong Provincial Hospital of Chinese Medicine, Guangzhou, Guangdong, China

**Keywords:** Traditional Chinese Medicine External Treatment, Conventional Western Medicine Treatment, Nocturia, Pittsburgh Sleep Quality Index, Response Rate

## Abstract

**Background:**

To assess the efficacy and clinical value of traditional Chinese medicine external treatment (TCMET) for older patients with nocturia.

**Materials and Methods:**

A systematic search was conducted in PubMed, Embase, Cochrane Library, Chinese National Knowledge Infrastructure (CNKI), Chinese Biological Medicine Database (CBM), VIP Chinese Science and Technology Journal Full-text Database (VP-CSJFD), and Wanfang database from their inception to September 30, 2024. Studies focusing on applying TCMET for older patients with nocturia were included. Primary outcome measures comprised the times of nocturia, Pittsburgh sleep quality index (PSQI) scores, and overall response rate. The extracted data was analyzed using Stata software (Version 17.0).

**Results:**

15 articles were included in this study (Figure-1), including 580 patients in the TCMET group and 318 patients in the conventional western medicine treatment (CWMT) group. The overall response rate after TCMET treatment in patients with nocturia was 94% [ES=0.94, 95%CI (0.91, 0.94)]. The number of nocturia episodes after treatment decreased by 2.39 times compared to before treatment [SMD=2.39, 95% CI (1.30, 3.48)]. TCMET treatment resulted in a 4.77-point decrease in patients’ PSQI scores from baseline [SMD=4.77, 95% CI: 1.67, 7.86]. Compared with CWMT, TCMET did not show significant superiority in improving overall response rate or PSQI scores (P0.05); however, it significantly reduced nocturnal voiding frequency (P0.05).

**Conclusion:**

According to the existing literature evidence, TCMET can reduce the times of nocturia and improve sleep quality in older patients with nocturia. It is an effective treatment for older patients with nocturia.

## Introduction

Nocturia is a condition characterized by the need to awaken from sleep to void urine.it is classified as nocturia when the volume of nocturnal urine exceeds one-third of the total daily urine output, or when there are over two episodes of nocturnal voiding per night [1]. A study shows that two-thirds of middle-aged and older adults are affected by this condition [2]. The incidence and prevalence of nocturia may vary depending on diagnostic criteria and geographic location. Its prevalence increases with age; among individuals aged over 60 and over 80 years, the incidence rates are 70% and 80%, [3]. In Shanghai, China, the prevalence of nocturia was reported to be 36.4%, with 47.4% in men and 52.6% in women [4]. A study conducted by Tikkinen et al. [5] found that among Finnish individuals aged 18 to 79 years, the prevalence of nocturia was 12% in men and 13% in women when defined as voiding at least twice per night, compared to 37% in men and 43% in women when defined as voiding at least once per night. According to Wang et al.’s research, 57.5% of individuals over 18 years old experienced nocturnal voiding once per night, while 24.7% experienced it twice per night [6]. Wen et al. [7] reported that among mainland Chinese men, the prevalence of nocturia, defined as voiding at least twice per night, was 30.8%. Therefore, nocturia affects a substantial patient population and has significant public health implications.

Although nocturia affects many patients, its etiology remains unclear. The etiology of nocturia is categorized into physiological and pathological factors [8]. Physiological factors include psychological influences, environmental stimuli, and unhealthy lifestyle habits, while pathological factors involve kidney and bladder disorders, hypertension, diabetes, and other systemic diseases [9]. Nocturia poses significant health risks to patients, disrupting nocturnal sleep and leading to symptoms such as fatigue, weakness, memory loss, and decreased quality of life [10].

In severe cases, it may also induce anxiety and depression. Western medicine or surgical interventions are used. Despite obtaining diverse Western medications, most are associated with prolonged treatment durations and potential side effects, which can impact patient adherence.

In contrast, Traditional Chinese Medicine (TCM) offers a unique approach that circumvents some limitations of Western medicine. TCM posits a profound understanding of the etiology and pathogenesis of nocturia in older adults, employing diverse therapeutic approaches [11]. According to TCM theory, the kidneys govern water metabolism, and normal micturition relies on the qi-transforming and steaming functions driven by kidney-yang.

In older adults, kidney-yang deficiency often leads to dysregulation of kidney opening-closing functions, resulting in nocturnal polyuria. Traditional Chinese medicine external treatment (TCMET), including acupuncture, moxibustion, and acupoint application, represents a distinctive therapeutic approach with diverse modalities. Although some studies have reported the clinical efficacy of TCMET for nocturia, high-level evidence-based medical validation remains limited. This study employs a meta-analysis to further evaluate the effectiveness and safety of TCMET in treating nocturia.

## Materials and Methods

### Literature Search Strategy

The literature search for this meta-analysis was conducted by the second and third authors (Jinhua Geng and Chunyan Ruan). A comprehensive search was performed across seven databases: PubMed, Embase, Cochrane Library, Chinese National Knowledge Infrastructure (CNKI), Chinese Biological Medicine Database (CBM), VIP Chinese Science and Technology Journal Full-text Database (VP-CSJFD), and Wanfang Database. The search timeframe for each database extended from their inception dates to September 30, 2024.

This meta-analysis employed combine Medical Subject Headings (MeSH) and free-text terms for comprehensive literature retrieval. Using PubMed as an exemplar database, the search syntax was constructed:("Nocturia"[Mesh] OR "Nocturia"[Title/Abstract] OR "Nocturnal polyuria"[Title/Abstract] OR "Nighttime urination"[Title/Abstract] OR "Bedwetting"[Title/Abstract] OR "Urinary frequency"[Title/Abstract]) AND ("Medicine, Chinese Traditional"[Mesh] OR "Traditional Chinese Medicine"[Title/Abstract] OR "TCM"[Title/Abstract] OR "Chinese herbal medicine"[Title/Abstract] OR "Acupuncture"[Mesh] OR "Acupuncture"[Title/Abstract] OR "Moxibustion"[Title/Abstract] OR "Acupoint application"[Title/Abstract] OR "Tuina"[Title/Abstract] OR "External therapy"[Title/Abstract]).

### Inclusion and Exclusion Criteria

Studies were eligible if they met the following criteria: (1) Participants were diagnosed with nocturia, defined as nocturnal urine volume exceeding one-third of total 24-hour output or ≥2 nighttime voiding episodes, with no restrictions on gender, age, ethnicity, or disease duration. (2) Interventions involved TCMET, including acupuncture, moxibustion, herbal acupoint application, auricular acupressure, or Tuina massage. (3) Control groups (if present) were limited to conventional Western medicine treatment (CWMT) or standard care, excluding any TCM-based interventions. (4) Primary outcomes included nocturnal voiding times, Pittsburgh Sleep Quality Index (PSQI) scores (assessed using Buysse DJ’s validated scale) [12], and the overall response rate. Efficacy was graded as: (a) Clinical cure (≤1 nighttime voiding), (b)Markedly effective (>50% reduction), (c) Effective (25-50% reduction), or (d) Ineffective (no improvement/worsening).

Studies were excluded if they met any of the following conditions: (1) studies with unavailable or insufficient data for extraction; (2) studies with a small sample size (fewer than 10 cases in either the experimental or control group); (3) low-quality publications; (4) non-English and non-Chinese literature; (5) duplicate publications of the same clinical study, in which case only the most complete and up-to-date version was kept; (6) case reports, literature reviews, systematic reviews, letters, and redundant publications; (7) preclinical studies involving cell or animal these criteria were strictly applied to ensure the validity and reliability of the included research.

### Data Extraction

The second and third authors (Jinhua Geng and Chunyan Ruan) conducted the study and extracted the relevant data. Where disagreements arose between the two authors, discussions were held with the fourth author (Qiaoli Lin) of this meta-analysis to reach a consensus. The extracted data included general information (first author, publication year, journal name) and clinical characteristics (patient sample size, age, follow-up duration). Clinical outcomes comprised nocturia times, PSQI scores, and the overall response rate.

### Literature Quality Evaluation

Following a meticulous review and in-depth analysis by the two authors (Jinhua Geng and Chunyan Ruan), studies that still exhibited significant discrepancies and lacked consensus were further evaluated by an additional author (Qiaoli Lin) to reach a final decision. To assess the quality of non-randomized controlled trials, we used the Methodological Index for Non-Randomized Studies (MINORS) scale, which provides a systematic evaluation framework [13]. Within this framework, studies scoring 12 points or higher were considered meeting the benchmark for high credibility. To evaluate the quality of randomized controlled trials, we applied the modified Jadad scale [14]. Studies scoring below 3 points were classified as having poor methodological quality, while those scoring above 3 points were deemed to show superior quality, thus warranting further attention and reference. This structured approach ensured rigorous and standardized quality assessment across all included studies, enhancing the reliability of our meta-analytic findings.

### Statistical Analysis

This meta-analysis employed Stata software (Version 17.0, StataCorp LLC, College Station, Texas, USA) for statistical analysis of the literature data. For studies without a control group, a single-arm meta-analysis was conducted to pool the response rates following TCMET treatment, using effect size (ES) and its 95% confidence interval (CI) as the effect measure (reflecting occurring specific events). For dichotomous data, the relative risk (RR) and its 95% CI were used as the effect measure. Weighted mean difference (WMD) and standardized mean difference (SMD), along with their 95% CI, applied to represent the effect sizes for continuous variables.

Heterogeneity among the included studies was assessed. If no significant heterogeneity was detected (P>0.1 and I²<50%), a fixed-effects model was used for pooled analysis; otherwise, a random-effects model was applied. In cases of substantial clinical heterogeneity, subgroup or sensitivity analyses were performed. A P-value <0.05 was considered significant.

When fewer than 10 studies were included, an asymmetric funnel plot was used to evaluate publication bias. If the number of included studies was less than 10, Egger’s test was employed to assess potential publication bias, with a P-value<0.05 showing its presence.

## Results

**Figure-1:**
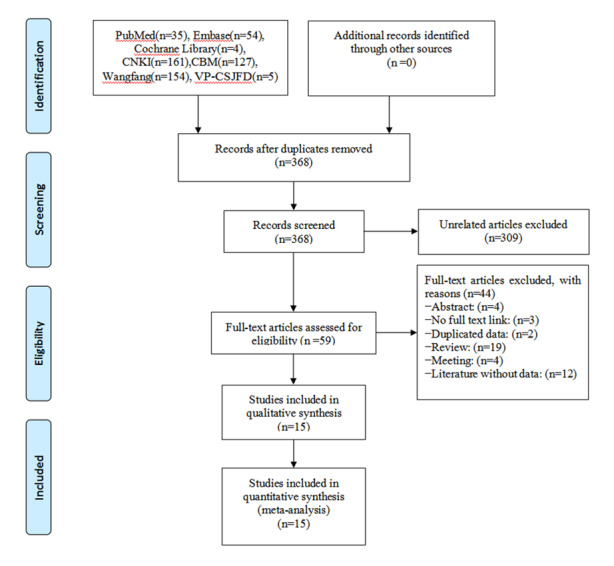
Figure 1. Flowchart of Literature Screening Process.

**Figure-2:**
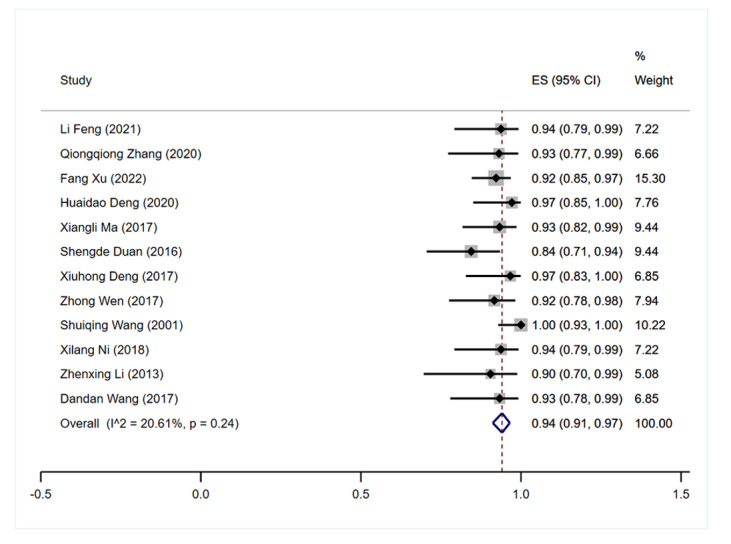
Figure 2. Forest Plot of Overall Response Rate for TCMET in Patients with Nocturia.

**Figure-3:**
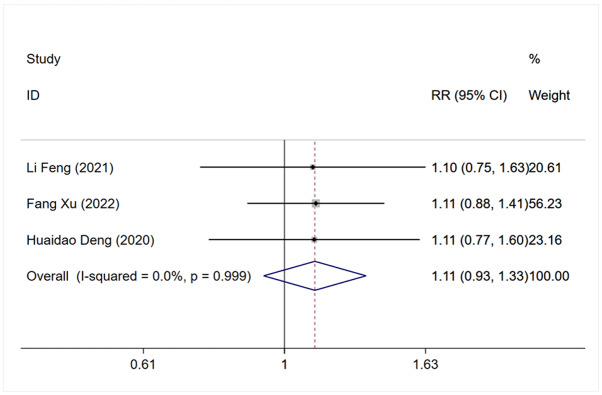
Figure 3. Forest Plot of Overall Response Rate Comparing TCMET and CWMT in Patients with Nocturia

**Figure-4:**
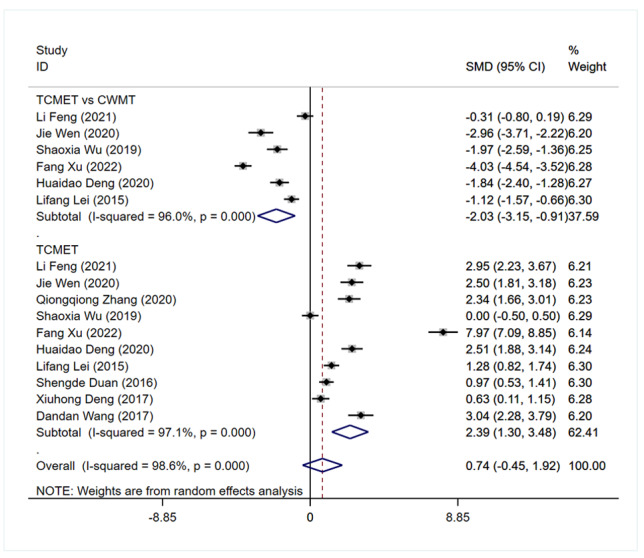
Figure 4. Forest Plot of Nocturia Frequency Comparing TCMET and CWMT in Patients with Nocturia.

**Figure-5:**
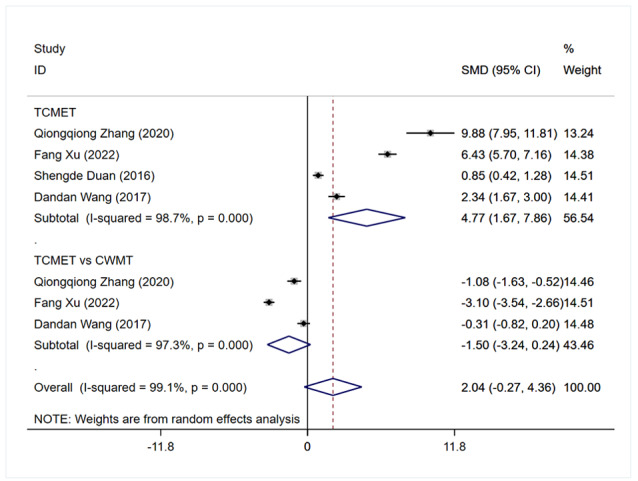
Figure 5. Forest Plot of PSQI Scores Comparing TCMET and CWMT in Patients with Nocturia.

**Figure-6:**
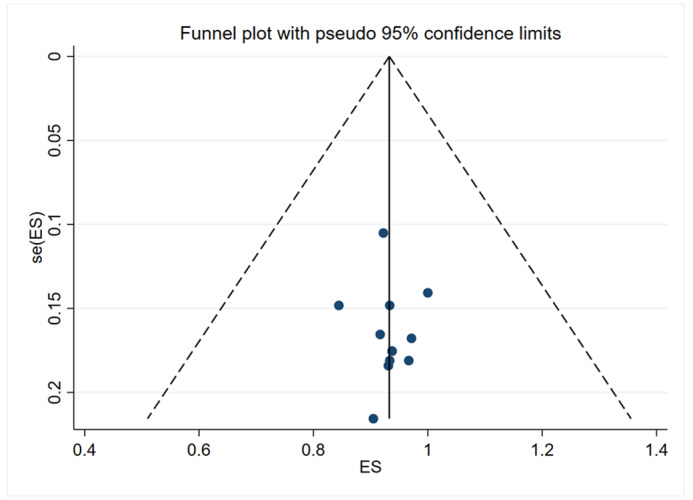
Figure 6. Funnel Plot of Overall Response Rate for TCMET in Patients with Nocturia.

**Figure-7:**
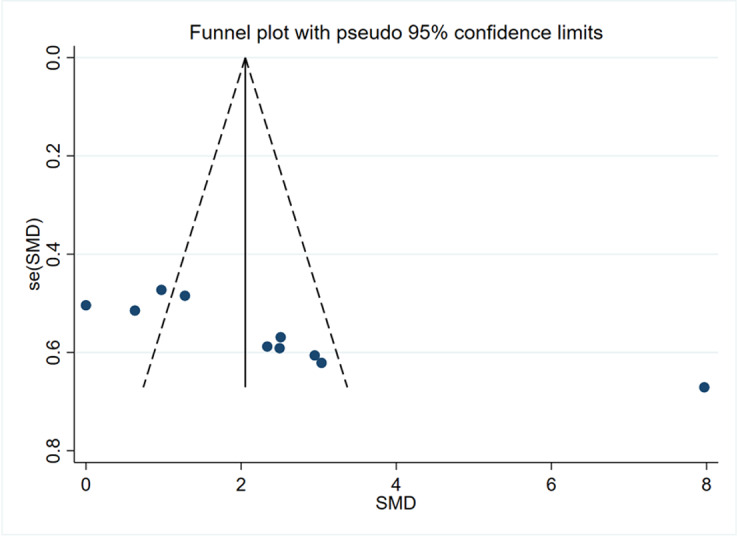
Figure 7. Funnel Plot of Nocturia Times for TCMET in Patients with Nocturia.

**Table:** Table1. Basic Characteristics and Quality Evaluation of the Included Literature

Author	Year	Country	Type of Study	Interventions		Sample Sizes		Gender (M/F)		Age (years)		Course of treatment	Outcomes	Jadad/Minors score
				OG	CG	OG	CG	OG	CG	OG	CG			
Li Feng ^[12] ^	2022	China	RCT	TCMET	CWMT	32	32	42/22		56-76		21d	①、③	4
Jie Wen ^[13] ^	2020	China	RCT	TCMET	CWMT	30	30	NA	NA	70.64 ± 10.89	70.21 ± 11.32	30d	①、②	4
Qiongqiong Zhang ^[14] ^	2020	China	RCT	TCMET	CWMT	29	28	13/16	11/17	59.414 ± 7.094	59.679 ± 7.870	60d	①、③、②	4
Shaoxia Wu ^[15] ^	2019	China	RCT	TCMET	CWMT	31	30	NA	NA	66.65 ± 12.01	70.20 ± 1.13	30d	①、②	3
Fang Xu ^[16] ^	2022	China	RCT	TCMET	CWMT	90	90	39/51	42/48	71.4 ± 2.7	72.7 ± 2.8	4 weeks	①、③、②	4
Huaidao Deng ^[17] ^	2020	China	RCT	TCMET	CWMT	35	35	18/17	20/15	68.5 ± 6.5	69.5 ± 4.5	5 months	①、③	4
Lifang Lei ^[18] ^	2015	China	RCT	TCMET	CWMT	44	43	29/15	29/14	NA	NA	21d	①、③	3
Xiangli Ma ^[19] ^	2017	China	Non-RCT	TCMET	NA	45	NA	31/14	NA	52-78	NA	22d	③	13
Shengde Duan ^[20] ^	2016	China	Non-RCT	TCMET	NA	45	NA	24/21	NA	65.8	NA	4 weeks	①、③、②	13
Xiuhong Deng ^[21] ^	2017	China	Non-RCT	TCMET	NA	30	NA	17/13	NA	70.91 ± 3.43	NA	14d	①、③	12
Zhong Wen ^[22] ^	2017	China	Non-RCT	TCMET	NA	36	NA	23/13	NA	69.3 ± 3.2	NA	30d	③	13
Shuiqing Wang ^[23] ^	2001	China	Non-RCT	TCMET	NA	50	NA	27/23	NA	55-60	NA	20d	③	14
Xilang Ni ^[24] ^	2018	China	Non-RCT	TCMET	NA	32	NA	13/19	NA	62	NA	22d	③	13
Zhenxing Li ^[25] ^	2013	China	Non-RCT	TCMET	NA	21	NA	13/8	NA	NA	NA	10 weeks	③	12
Dandan Wang ^[26] ^	2017	China	RCT	TCMET	CWMT	30	30	16/14	17/13	NA	NA	4 weeks	①、③、②	4

TCMET: traditional Chinese medicine external treatment; CWMT: Conventional western medicine treatment; OG: Observation group; CG: Control group; ①: Times of nocturnal urine; ②: Pittsburgh sleep quality index (PSQI) scale score; ③: Overall response rate.

### 1. Literature Screening and Procedures

In this meta-analysis, 540 articles were identified. After removing duplicates, 368 articles remained. Following abstract screening, 59 articles were excluded because of their failure to meet the inclusion criteria. After excluding studies with outdated publication dates or insufficient extractable data, 15 articles [15][16][17][18][19][20][21][22][23][24][25][26][27][28][29] were included. Figure-1 presents a detailed flowchart of the literature selection process.

### 2. Basic Characteristics and Quality Evaluation of Included Literature

The studies included in this meta-analysis were all published in Chinese between 2001 and 2022. Among these, seven were randomized controlled trials (RCTs), two of which had Jadad scores below 4 [18][21], while the remaining RCTs scored 4 or higher. In these trials, the experimental group received TCMET for fever intervention, whereas the control group received CWMT. The other eight studies were non-randomized controlled trials with self-controlled before-and-after designs, all scoring ≥12 on the MINORS scale, with interventions also involving TCMET in Table-1. Overall, the methodological quality of the included studies was acceptable.

### 3. Meta-analysis Results

#### 3.1. The Overall Response Rate of Nocturia Treatment

Twelve studies [15][17][19][20][22][23][24][25][26][27][28][29] reported the overall response rate following TCMET in patients with nocturia. A pooled analysis of 475 nocturia cases treated with TCMET showed low heterogeneity among the included studies (I²=20.61%, P>0.10). The meta-analysis revealed a high overall response rate of 94% [ES=0.94, 95% CI (0.91, 0.97)], showing significant therapeutic efficacy of TCMET for nocturia (Figure-2). Three studies [15][19][20] compared the overall response rate between TCMET and CWMT, involving 157 nocturia patients across both groups. Heterogeneity analysis showed no significant variation (I²=0.0%, P>0.10). The meta-analysis showed no significant difference in efficacy between TCMET and CWMT [RR=1.11, 95% CI (0.93, 1.33), P >0.05, Figure-3].

### 3.2. The Nocturia Times of Nocturia Treatment

Ten studies [15][16][17][18][19][20][21][23][28] reported nocturia episode times before and after TCMET in patients with nocturia. The meta-analysis of data from 396 nocturia patients treated with TCMET showed substantial heterogeneity among the included studies (I²=97.1%, P<0.10). Results showed that post-treatment nocturia episode times were reduced compared to baseline measurements, with an SMD of -2.39 [95% CI (-3.48, -1.30), P<0.05].

Six comparative studies [15][16][18][19][20][21] evaluated nocturia times between TCMET and CWMT groups, involving 262 patients in the TCMET group and 260 patients in the CWMT group. Significant heterogeneity was observed across these studies (I²=96.0%, P<0.10). The meta-analysis revealed superior efficacy of TCMET, with treated patients showing lower nocturia times compared to those receiving CWMT [SMD=-2.03, 95% CI (-3.15, -0.91), P<0.05, Figure-4].

### 3.3. The PSQI Score of Nocturia Treatment

Four articles [17][19][23][29] reported PSQI scores before and after TCMET in patients with nocturia. 194 nocturia patients were treated with TCMET. Heterogeneity tests conducted on the four included articles revealed substantial heterogeneity (I²=98.7%, P<0.10). Meta-analysis results indicated PSQI scores were reduced by 4.77 points following TCMET treatment compared to baseline [SMD=-4.77, 95% CI (-7.86, -1.67), P<0.05].

Three articles [17][19][29] compared PSQI scores after TCMET and CWMT in nocturia patients. 149 patients received TCMET, while 148 patients received CWMT. Heterogeneity tests performed on these three articles also showed high heterogeneity (I²=99.1%, P<0.10). Meta-analysis results showed no significant difference in PSQI scores between TCMET and CWMT groups [SMD=-1.50, 95% CI (-3.24, 0.24), P>0.05, Figure-5].

### 4. Sensitivity Analysis and Regression Analysis

In this study, significant heterogeneity was observed in two outcome measures (nocturia frequency and PSQI scores). Therefore, subgroup analysis and regression analysis were conducted to explore the sources of heterogeneity.

Using the one-by-one removal method, no primary source of increased sensitivity was identified. After excluding any single study, the meta-analysis results for nocturia times and PSQI scores remained consistent with those obtained before exclusion. Thus, the results of this meta-analysis were deemed reliable. To further investigate the sources of heterogeneity, regression analysis was performed based on factors such as treatment duration, age, and study type. However, no primary source of heterogeneity was detected through this regression analysis.

### 5. Publication Bias

Among the three outcome measures—the nocturia times, PSQI score, and overall response rate—the number of articles included in the overall response rate (12 articles) and nocturia times (10 articles). For these outcomes, publication bias was assessed by inspecting the symmetry of the funnel plot. The results showed that the points on the funnel plots for these two outcome measures were scattered and exhibited incomplete symmetry, suggesting some publication bias (Figures-6 and -7 ). For the PSQI scale score, only 4 articles were included, which is fewer than 10. Here, publication bias was evaluated using Egger's test, and the results suggested no significant publication bias among the included articles (P>0.05).

## Discussion

In this study, we found that the overall response rate after TCMET treatment in patients with nocturia was 94%, the times of nocturia episodes decreased by a factor of 2.39 compared to before treatment, and the PSQI score decreased by 4.77 points relative to pre-treatment levels. These findings suggest TCMET is effective for treating nocturia and can improve nocturia symptoms.

Therefore, TCMET may serve as an alternative treatment option for patients who do not respond to behavioral therapy or pharmacological interventions.

Interestingly, while TCMET significantly reduced nocturnal voiding frequency compared with CWMT, it did not show superiority in improving overall response rate or PSQI scores. This discrepancy suggests that nocturia frequency may be a more sensitive outcome measure for capturing the clinical benefit of TCMET, whereas global response rates and sleep quality indices may be influenced by multiple confounding factors.

Nocturia is primarily managed, with behavioral therapy and drug therapy being the main approaches to reduce nocturnal urination frequency and improve patients’ quality of life based on its underlying pathophysiology. Behavioral therapy serves as an appropriate first-line treatment for nocturia, regardless of its etiology [30].

Specific strategies include reducing fluid intake (especially caffeine and alcohol) at least 2 hours before bedtime, limiting total daily fluid consumption to less than 2L, ensuring complete bladder emptying before sleep, shortening sleep duration to enhance sleep efficiency, increasing physical activity including pelvic floor exercises, maintaining an optimal night-time rest environment by keeping warm and promoting blood circulation, reducing dietary salt intake, managing body weight for overweight patients, and elevating the lower limbs at bedtime for patients with congestive heart failure or chronic venous insufficiency-induced lower limb edema to minimize water retention. However, since nocturia often progresses despite lifestyle modifications, medical intervention is considered as a subsequent step [31][32].

After a poor response to behavioral therapy, drug therapy is considered the next step. Desmopressin [33][34] is the only drug approved for treating nocturia and is indicated for patients with normal average bladder capacity who produce excessive nocturnal urine output. A systematic review shows that desmopressin acetate is effective in improving nocturia symptoms; however, its most common adverse effects include hyponatremia and headache [35].

Diuretics are also used to manage nocturia, with the primary mechanism being reducing salt and water load in the body before bedtime [36]. Non-steroidal anti-inflammatory drugs (NSAIDs), selective α1-adrenergic receptor antagonists, and 5α-reductase inhibitors have been employed for nocturia treatment, but their clinical efficacy remains limited [37]. Sedatives have also been reported as a potential option for treating nocturia [38]; however, their use in older patients should be approached with caution because of side effects such as cognitive dysfunction, drug dependence, and rebound insomnia.

Besides the aforementioned Western medicine treatments, TCM such as Jinkui Shenqi Pills, Yiqi Gushen Decoction, Shuoquan Pills, and others also show specific efficacy for nocturia [39][40][41]. However, TCM decoctions and medications involve complex preparation requirements.

Most TCM decoctions have an unpleasant taste and require long-term administration, which may pose an additional inconvenience for patients with underlying diseases. Oral conventional TCM formulations are even less convenient in such cases. TCM pills exhibit a slower onset of action and cause a longer treatment duration, making them less suitable for individuals requiring rapid symptom relief. Since both TCM decoctions and pills are absorbed through the gastrointestinal tract, their long-term use might lead to adverse effects on the body.

At present, there is no specific clinical treatment for nocturia. Because of the limitations imposed by degenerative changes in various organs of the older adults population, clinical practice relies on comprehensive symptomatic treatments, including behavioral training, bedtime administration of diuretics to promote water and sodium excretion, oral medications for relieving bladder hyperactivity, and drugs for benign prostatic hyperplasia.

However, patients often exhibit poor compliance with behavioral training, while drug treatments are associated with significant toxic side effects, limiting their clinical application. Therefore, improving nocturia in older adults has become a key research topic for clinicians.

Besides conventional drug therapy, TCMET offers an alternative approach. This meta-analysis retrieved TCMET interventions for nocturia, including acupuncture, moxibustion, external application of traditional Chinese medicine, auricular point pressing bean therapy, and manual massage. Conventional acupuncture, Xingnao Gu needling, scalp acupuncture, and electroacupuncture have shown specific efficacy for nocturia in middle-aged and older adults. Acupoints such as Sanyinjiao (SP6), Shenshu (BL23), Baihui (GV20), Zhongji (CV3), Guanyuan (CV4), Wanshu (BL21), Qugu (ST28), Transverse Bone (EX-CN7), and Taixi (KI3) are selected [15][22][23][26].

Moxibustion therapy is widely used, with methods such as mild moxibustion, meridian flow injection moxibustion, heat-sensitive moxibustion, warm acupuncture, medicinal cake moxibustion, paving moxibustion, and Du moxibustion achieving some efficacy [16][17][18][24][25][27]. Manipulative massage focuses on conditioning the Ren and Du meridians, selecting critical acupoints such as Mingmen (GV4) and Guanyuan (CV4) to improve nocturia in middle-aged and older adults based on the same-meridian acupoint theory [28]. Acupoint application involves selecting acupoints such as Shenshu (BL23), Pishu (BL20), Shenque (CV8), Yongquan (KI1), and Guanyuan (CV4), combined with the use of Shuoquan Pills, which has shown specific efficacy [19][20][21][29].

However, because of the diversity of TCMET approaches, the specific TCMET interventions used by different investigators vary, making it challenging to achieve broader recognition of its efficacy in treating nocturia.

This meta-analysis integrated 15 articles and confirmed that TCMET is an effective treatment for nocturia symptoms in patients, strengthening the evidence base for its efficacy. However, TCMET did not show significant superiority over CWMT in terms of overall response rate or PSQI score improvement (P>0.05). Only in reducing nocturia frequency was TCMET more effective than CWMT (P<0.05).

Nocturia frequency is influenced by various causes, with one key mechanism being arginine vasopressin secretion deficiency. Anti-diuretic desmopressin acetate treatment addresses nocturnal arginine vasopressin secretion deficiency [33]. Desmopressin acetate regulates water retention in the human body by enhancing osmotic driving force, promoting water reabsorption and trans-cellular water transport in the kidney, leading to increased urine concentration and reduced urine output. Antimuscarinic and alpha-blockers are also important in treating overactive bladder and benign prostatic hyperplasia, achieving significant clinical efficacy [31][42].

While pharmacological treatments show apparent effects on nocturia, their superiority or inferiority compared to TCMET in terms of treatment response rate and PSQI score improvement remains unclear because of the limited number of studies comparing TCMET and CWMT (only three articles were included in this analysis). However, regarding nocturia times, TCMET showed greater effectiveness than CWMT, as evidenced by six articles, providing strong support for its superiority in improving nocturia frequency. Therefore, TCMET can serve as an alternative treatment option for patients who do not respond to behavioral or pharmacological therapies.

The high heterogeneity observed in nocturia frequency and PSQI outcomes (I²>90%) likely reflects differences in intervention types (acupuncture, moxibustion, acupoint application), treatment duration, and patient populations. Future RCTs with standardized protocols are required to reduce variability and strengthen the evidence base.

Although this study confirms the effectiveness of TCMET in treating nocturia, several limitations should be acknowledged. First, among the 15 studies included in this meta-analysis, not all were randomized controlled trials, which limits the overall strength of evidence supporting the conclusions. Second, TCMET encompasses a variety of external treatment methods, and it is challenging to ensure the efficacy of each specific method because of its diversity.

The results for the PSQI score and nocturia frequency exhibited high heterogeneity. Despite conducting sensitivity analysis and regression analysis, no definitive explanation for the high heterogeneity was identified, which may affect the reliability of the conclusions to some extent. Given these limitations, it is recommended that future large-scale, well-designed, randomized controlled trials be conducted to further validate the findings.

## Conclusion

TCMET demonstrates potential effectiveness in treating nocturia, particularly in reducing nocturnal voiding frequency. However, its superiority over conventional treatments in terms of overall response rate and sleep quality remains inconclusive. However, because TCMET has many methods, which TCMET has better efficacy has yet to be clarified, and subsequent investigators can further explore this aspect to select more valuable TCMET for patients.

The current meta-analysis is subject to a number of important limitations. Although it synthesized results from 15 separate studies, the evidence base is weakened by the small number of high-quality randomized controlled trials, the remainder comprising mostly studies with limited sample size or without randomization. Second, a high degree of heterogeneity was found for key outcomes, especially nocturia frequency and PSQI scores, with I² values exceeding 90 percent; this variability likely arises from differences in the type of intervention employed (for example, acupuncture, moxibustion, or point application), treatment length, point selection, differences in baseline patient characteristics, and the definitions of outcomes. Third, the heterogeneous TCMET modalities do not permit us to single out the most effective technique, and so the applicability of the findings is restricted.

Fourth, the majority of studies appeared in Chinese-language journals, which poses a risk of language and publication bias; studies with neutral or negative findings may have been omitted.

Fifth, there was a lack of common reporting conventions for outcomes, with the notion of "response rate" defined variously, complicating direct meta-analysis. Sixth, almost all studies limited follow-up to a relatively brief duration, which hinders any assessment of durability or safety over the longer term. Lastly, factors that might confound results, such as comorbid conditions, concurrent pharmacotherapy, and lifestyle variables, were inconsistently reported, and in many cases not adjusted for, thereby casting doubt on the precision of the treatment effect estimates.

## Conflict of Interest

All authors declare they have no conflicts of interest regarding publishing this manuscript.
